# Preferred sound groups of vocal iconicity reflect evolutionary mechanisms of sound stability and first language acquisition: evidence from Eurasia

**DOI:** 10.1098/rstb.2020.0190

**Published:** 2021-05-10

**Authors:** Johannes Dellert, Niklas Erben Johansson, Johan Frid, Gerd Carling

**Affiliations:** ^1^ Seminar für Sprachwissenschaft, Universität Tübingen, Wilhelmstraße 19, 72074 Tübingen, Germany; ^2^ Center for Languages and Literature, Lund University, Helgonabacken 12, 223 62 Lund, Sweden; ^3^ Lund University Humanities Lab, Lund University, Box 201, 221 00 Lund, Sweden

**Keywords:** sound evolution, vocal iconicity, phonology, typology, language evolution, first language acquisition

## Abstract

In speech, the connection between sounds and word meanings is mostly arbitrary. However, among basic concepts of the vocabulary, several words can be shown to exhibit some degree of form–meaning resemblance, a feature labelled vocal iconicity. Vocal iconicity plays a role in first language acquisition and was likely prominent also in pre-historic language. However, an unsolved question is how vocal iconicity survives sound evolution, which is assumed to be inevitable and ‘blind’ to the meaning of words. We analyse the evolution of sound groups on 1016 basic vocabulary concepts in 107 Eurasian languages, building on automated homologue clustering and sound sequence alignment to infer relative stability of sound groups over time. We correlate this result with the occurrence of sound groups in iconic vocabulary, measured on a cross-linguistic dataset of 344 concepts across single-language samples from 245 families. We find that the sound stability of the Eurasian set correlates with iconic occurrence in the global set. Further, we find that sound stability and iconic occurrence of consonants are connected to acquisition order in the first language, indicating that children acquiring language play a role in maintaining vocal iconicity over time.

This article is part of the theme issue ‘Reconstructing prehistoric languages'.

## 1. Introduction

Human speech production involves an ability to form a range of distinct phonemes, which are a precondition for spoken language. The evolution of speech production in pre-historic language can be approached in various ways. Studies in the evolution of articulation can contribute to how sound distinctions emerged [1], cross-linguistic typologies of sound systems can be used to classify sounds and systems into types [2], and reconstructions by the comparative method can indicate how sound systems evolve over time [3]. In addition, first language acquisition (L1) can be viewed in terms of evolution [4,5].

The basis for sound evolution is the articulatory mechanisms of speech production, studied in the disciplines of phonetics and phonology [6]. Cross-linguistic typologies, aiming at understanding the processes leading to global phonological diversity, rely on basic features of articulation [7] and in a typological model [8], synchronic regularities are a result of diachronic trajectories of change [9]. Over time, sound systems are continuously modified, a process identified as sound change. Various mechanisms govern this process, most importantly articulation, which impacts the directionality of change [10,11].

Further, sound evolution may be connected to articulatory mechanisms of L1. This idea, known as the child-language theory by Jakobson [4,12], is continued in the theory of preference laws in phonological change [13]. The approach connects evolution and typology with acquisition, implying that a specific structure is preferred with respect to some parameter in a specific situation, following the principles that [5]:

(1) the more languages show a property cross-linguistically, the more preferred it is;
(2) the earlier and quicker it is acquired in L1, the more preferred it is;
(3) the longer it takes to become lost in aphasia, the more preferred it is.

Preference laws build on the inversion of consonantal strength, a relational measure defined as the degree of deviation from an unimpeded airflow, and the sonority hierarchy, which organizes phonemes according to their acoustic energy [6,14]. Thus, the theory sees language change as striving towards a maximal contrast in the syllable, with the strongest possible consonant and a maximally sonorous vowel [15] (table 1). Studies in L1 indicate that the variation between individual children may be substantial [16], but a general acquisition order of speech production can be supported by data from normal and impaired children [16–20]. This order can also be connected to phonological simplifications in aphasia patients [21].

**Table 1.** The scale of consonantal strength and sonority, as presented in the theory of preference laws [5,13,15].

Increasing consonantal strength
voiceless stops
voiced stops
voiceless fricatives
voiced fricatives
nasals
lateral liquids
central liquids
glides/approximants
high vowels
mid vowels
low vowels
Increasing sonority

The notion of vocal iconicity or sound symbolism, a resemblance-based mapping between form and meaning, has a long history in linguistic literature [22]. To Saussure [23], the notion of an arbitrary connection between form and meaning of the linguistic sign was a precondition to his general theory of language. This notion of arbitrariness was questioned by Jespersen [24] and Jakobson [4,25], who observed that sound symbolism is not just an important feature of language itself; it is also a vital part of acquiring a language [26,27].

An important aspect of iconicity—in both speech and signs—is the role it may have played in pre-historic language. Whereas signs have a natural tendency to be motivated by iconicity, indexicality or systematicity [28,29], the connection is less evident for speech [30–32]. The discussion is about whether speech is fundamentally iconic or arbitrary and whether signs preceded speech in pre-historic language or not [33,34]. One theory argues that pre-historic language initially consisted of iconic signs, which were later successively replaced by conventionalized symbols, giving rise to arbitrariness [35,36]. A competing theory argues that speech perforce is arbitrary, manifests itself by convention [30,37], and therefore must have been arbitrary from the start. An often-invoked argument is the communication signals of vervet monkeys and other animals, which are supposed to be arbitrary [38]. However, communication signals by non-human primates may be iconically or indexically motivated [39], and there is evidence that chimpanzees can map white/bright to high-pitched sounds and black/dark to low-pitched sounds [40], so the question remains open.

Recent empirical research, using experiments and large datasets, challenges the notion of the fundamental arbitrariness of speech. Vocal iconicity is central to various aspects of language processing and communication [22,41]. Several studies, using large, cross-linguistic datasets and computational modelling, indicate that a substantial part of basic vocabulary (the universally common, most frequent and salient part of vocabulary) is non-arbitrarily motivated [42–44].

Our paper deals with vocal iconicity and the evolutionary mechanisms of speech production. A fundamental problem here is the cross-linguistic occurrence of vocal iconicity in spite of language change. In a Neogrammarian model [45,46], sound change is inevitable, rule-based (including conditions and constraints of change) and governed by articulatory principles [3]. The Neogrammarians did not specifically mention arbitrariness, but a prerequisite to the model is that sound change is ‘blind' to the meaning of words. Even though the Saussurean notion of the arbitrariness was questioned by later scholars, they did not address the problem of iconicity in relation to inevitable and meaning-blind sound change, forming the baseline for our study. If speech is fundamentally arbitrary and agreed upon by convention, how come that vocal iconicity emerges in core parts of the vocabulary of all languages? Reversely, if speech is fundamentally iconic, how come that vocal iconicity is not destroyed by a meaning-blind sound change?

## 2. Theory

Since a vital part of basic vocabulary remains iconically motivated [42,44], we assume that the emergence and preservation of vocal iconicity is a process that is integrated with core parts of the sound evolution process. To make a cross-linguistic investigation feasible, we restrict ourselves to basic vocabulary, focusing on concepts which have demonstrated sound–meaning associations from a cross-linguistic perspective, together with concepts that have not done so [42–44].

**Figure 1.**
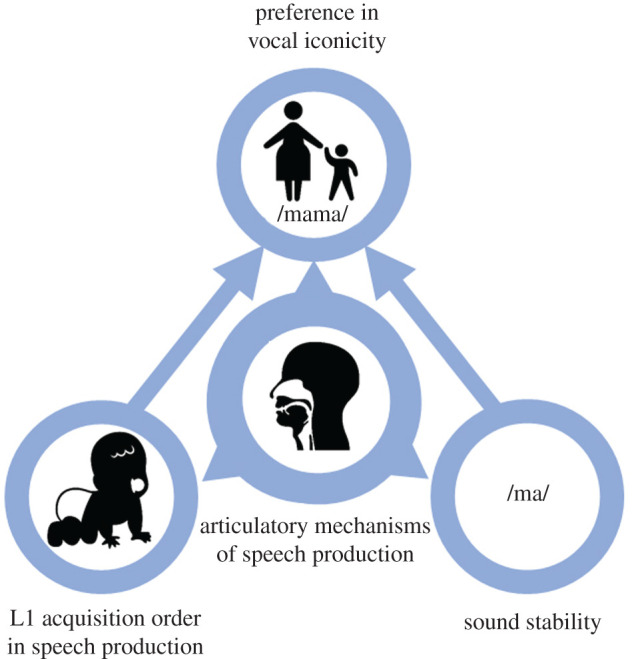
Representation of our model of emergence of vocal iconicity in relation to articulatory mechanisms of speech production, sound stability and sound preference in first language acquisition.

Using the preference theory as a backdrop [4,5,13], we assume that sound preference, controlled by articulatory mechanisms of speech production, affects sound evolution over time. Here, we assume a gradual decrease in stability of sound groups along the parameters outlined by the preference theory (relying on the sonority scale), roughly following the principle that consonants range in preference from front to back (strongest to weakest), and vowels range from back to front/mid (weakest to strongest). In addition, we assume that articulation affects L1 along these parameters. Due to the role of vocal iconicity in L1 [47], we assume that vocal iconicity, by iconic preference, is encapsulated in the general sound evolution process by means of mechanisms of articulation. Sound groups that are more salient in evolution as well as preferred in acquisition correlate with sound groups that are overrepresented in iconic associations (figure 1). Using the model of phonemic feature hierarchies [7], we identify five basic feature classes, matching the preference theory. These are: (1) place of articulation, (2) manner of articulation, (3) voicing, (4) openness and (5) backness (table 2). By means of these parameters, our different datasets can be compared and evaluated to test our theory. Our study is confined to contemporary, cross-linguistic data. One of our datasets has a cross-linguistic, global coverage, picking one language per family (iconic occurrence); the other dataset is restricted to one continent (Eurasia), including data from 21 families (sound evolution). We admit that the difference in data coverage is a shortcoming, but global data on sound evolution was not available to us. However, even though the model of uniformitarianism [48] is becoming increasingly questioned (see other papers of this volume), we assume that our limited sample still provides a good approximation to a general model of the mechanisms of speech production in pre-historic language and the general trajectories of language change underlying the emergence of iconicity [31,34].

**Table 2.** Scheme of sound groups, organized by classes [7] and defined as earlier and later in first language acquisition. The distinctions follow the babbling period [19] and are relative notions, not specifically distinguishing absolute ages of acquisition. They were first identified by Jakobson [4] and have been verified by different methods (diary method, day-by-day recording) in various languages (see electronic supplementary material, S3).

	sound classification		first language acquisition		
	main type	feature class^a^	earlier	later	source
1	consonants	place of articulation	labial, alveolar	palatal, velar, glottal	[16,17]
2	consonants	manner of articulation	stop	continuant	[16]
3	consonants	voicing	voiceless	voiced	[17]
4	vowels	openness	low	high, mid	[18]
5	vowels	backness	back	front, central	[18]

^a^the table contains feature classes of sound groups that are distinguished in our data [7]. Other observed groups are labial nasals (earlier)–other nasals (later) [16] and unaspirated stops (earlier)–aspirated stops (later) [20].

## 3. Model, method and data

Our model is empirical and quantitative, considering sound *evolution* rather than sound *change.* For that purpose, we use cross-linguistic data and a method that can be applied equally to several families, independently of the degree to which they are covered by the comparative method. The model does not consider conditions and constraints of sound change, which is a complex process involving factors such as metathesis, merger, loss, lenition, epenthesis, syncope and apocope [3].

Our linear regression model correlates three different datasets:

(1) (SSt) = a new dataset of sound group stability data, based on all International Phonetic Alphabet (IPA) transcriptions from the NorthEuraLex 0.9 database, i.e. words for 1016 concepts across 107 languages from 21 language families of Northern Eurasia.
(2) (Ico) = an existing, published dataset of iconic preference for 344 basic concepts, covering one language each from 245 families [44]. Lexemes have been coded by sound groups relevant to acoustic and articulatory mechanisms, as well as to vocal iconicity values.
(3) (L1) = a small reference dataset in the form of a matrix of sound groups by feature class, identified as earlier or later in L1.

### (a) Sound stability

In order to substantiate our assumption that the appearance of iconic form–meaning mappings may in part be explained by the higher overall stability of certain sound groups against phonetic change, we propose a way to estimate sound group stability values (SSt) from a cognacy-annotated phonetic form database and apply it to NorthEuraLex 0.9 [49]. We modify the existing code for information-weighted sequence alignment (IWSA) [50] to support the IPA segmentation underlying the iconicity dataset (Ico) [44].

Based on this re-segmentation, we first run the relevant script from the IWSA code on NorthEuraLex 0.9, in order to infer sound similarity scores for sequence alignment of word pairs. To decide which word pairs to align, we re-use the previously published automated homologue judgements returned by the IWDSC method [50]. Initially, we considered building on preliminary results of cognacy annotation based on the available literature. However, published coverage of etymologies for the relevant families remains both incomplete and uneven, and mixing partial high-quality cognacy annotations with low-quality automated judgements for the rest of the data could easily lead to difficult-to-handle statistical biases. We are aware that the automated annotations are of much lower quality than would be achievable for large parts of our data (e.g. for Indo-European and Uralic), but from the statistical point of view, the data quality issues can simply be treated as high noise levels, which will not distort results much as long as the noise is equally distributed across the entire dataset.

The central idea of our sound group stability estimates is to count how many instances of sounds from the respective group in the database are aligned to sounds from the same group in pairs of homologue forms. This provides an empirical answer to the question of how commonly phonetic evolution leads to sounds losing their group-defining properties. However, two major biases inherent in a simple tally of the aligned sound pairs in the relevant alignments need to be avoided.

The first problem is that the language pairs represent different durations of phonetic divergence. If some sound groups are overrepresented in a group of closely related languages in the database (such as the Slavic languages), simply counting all the aligned sound pairs involving that group will overestimate stability. In order to avoid this bias, we weight the counts by the average stability of sounds across all homologue pairs for the language pair in question. For instance, for Finnish and closely related Karelian, 779 word pairs were automatically detected to be homologues, and 5513 segment pairs were aligned in total by IWSA. Out of these, 3968 segment pairs consisted of identical IPA symbols. Ultimately, the stability value of Finnish towards Karelian was computed to be as high as 0.791, i.e. only one in five sounds will be different between etymologically related words from the two languages. To compute stability values for a sound group, each relevant sound pair from alignments of words from this language pair was therefore counted as only 20.9% of a full sound pair. For Tundra Nenets, the Uralic language which is most distant from Finnish according to the stability measure, the equivalent value is at 74.6%, i.e. three out of four aligned sounds are non-identical. A sound which remains identical between Finnish and Tundra Nenets is thus counted more than three times as much as a sound which merely remained identical in Finnish and Karelian.

The second bias to avoid is caused by the use of dictionary forms as opposed to stems which would be the most natural etymological comparanda. Mitigating the effects of this property of lexicostatistical databases is the main motivation for information weighting as introduced by Dellert & Buch [51], of which Dellert [50] represents the most recent version. The information content quantifies for each position in an alignment how surprising the segments are in their current context, automatically leading to low values for recurring inflectional material such as infinitive endings. We simply multiply the weight by which each instance in an alignment is counted by its information content. In sum, each segment pair count is weighted by the product of the information content and the overall rate of phonetic replacement between the two relevant languages.

We compute the SSt values for each sound group by dividing the weighted count of the identical or in-group alignments by the total weighted count of alignments involving sounds from a sound group. The exact mathematical statement of the SSt measure with all of its components can be found in the electronic supplementary materials (S1), and the implementation is openly available as part of our code release.^1^

### (b) Iconic value

In order to investigate whether there is a correlation between the stability of a sound group and the frequency of its usage in iconic sound–meaning associations, we need to build on reliable and comparable stability and iconicity values for articulatory and acoustic features. For this, we re-use the iconicity values from Erben Johansson *et al*. [44], where the sampled data were phonetically transcribed and the segments were grouped according to salient articulatory parameters and distinctive acoustic features relevant for studying iconicity. Proportional over- and underrepresentations of each sound group for each concept could then be estimated. These were then transformed into odds ratios (OR) in order to make sound groups with different levels of granularity comparable (e.g. unrounded-rounded vowels versus high-mid-low vowels). A region of practical equivalence (ROPE) was then defined around the null effect of no under- or overrepresentation. Noteworthy (strong) overrepresentations were defined as a 25% increase of the OR which also had the 95% credible intervals for the OR falling completely outside the ROPE. Noteworthy (weak) overrepresentations were defined as a 25% increase of the OR which also had the 95% credible intervals for the OR excluding zero and the median of posterior distribution was outside the ROPE. Altogether, this produced very conservative estimates of the degree of under- or overrepresentation. Two hundred and twenty-five combinations of sound groups and concepts were judged to be iconically overrepresented which corresponded to only approximately 1.3% of all possible combinations. In order to ensure comparability, we re-use the sound group categorization from this study for computing the stability values (SSt). The only differences are that voiced glottals, voiceless nasals, voiceless laterals, voiceless vibrants and low front rounded vowels had to be excluded due to data sparsity for these sound groups in the NorthEuraLex database. All sound–meaning comparisons with iconicity values lower than 1 (i.e. underrepresentations of sounds) were removed because most of these are redundant mirror images of overrepresentations (an overrepresentation of rounded vowels also leads to an underrepresentation of unrounded vowels) and would thus skew the comparison to sound stability.

### (c) Earlier and later in first language acquisition

To contrast our data of SSt and Ico against preference in L1, we compile a smaller reference dataset from various sources (electronic supplementary material, S3). First, we list hierarchical chains for sound groups to be acquired earlier or later in first language acquisition [4]. Second, we identify phonemic feature classes (place and manner of articulation, voicing, openness and backness) [7] that can be applied to our sound groups (electronic supplementary material, S2), matching the preference theory. Finally, we scan the literature on acquisition order in the first language to verify these feature classes and compile a matrix, which merges the suggested chains into two relative types, ‘earlier’ and ‘later’ (with no specific reference to age). We use mainly data following the babbling period (table 2).

To arrive at our combined dataset (electronic supplementary material, S4), we start from the iconic value dataset (Ico), which contains 18 576 rows of concepts in combination with individual sound groups, based on the lexical data. The calculation of iconic value in the original dataset is Bayesian and includes a confidence interval. We use the centre of the confidence interval as the prototypical value. To this data, we add the sound stability values of the SSt dataset for each row (col K). Thereupon, we add the reference data from the L1 matrix (table 2) for each sound group present here. We were not able to identify safely a potential coding ‘earlier’ or ‘later’ for all sound groups of our dataset Ico, which means that these columns contain several empty cells. However, all sound groups containing the relevant phonetic feature of the L1 matrix (table 2), regardless of granularity level, are coded. For example, stop consonants include all stops (from the five-level distinction of the nasal, stop, continuant, vibrant, lateral sound groups), but also voiced nasals, voiced stops, voiced continuants, voiced vibrants and voiced laterals (from the ten-level distinction of the nasal, stop, continuant, vibrant, lateral sound groups with voiced and voiceless distinctions). We analyse the joint data by linear regression tests, using R (electronic supplementary material, S5; see below).

## 4. Results

**Figure 2.**
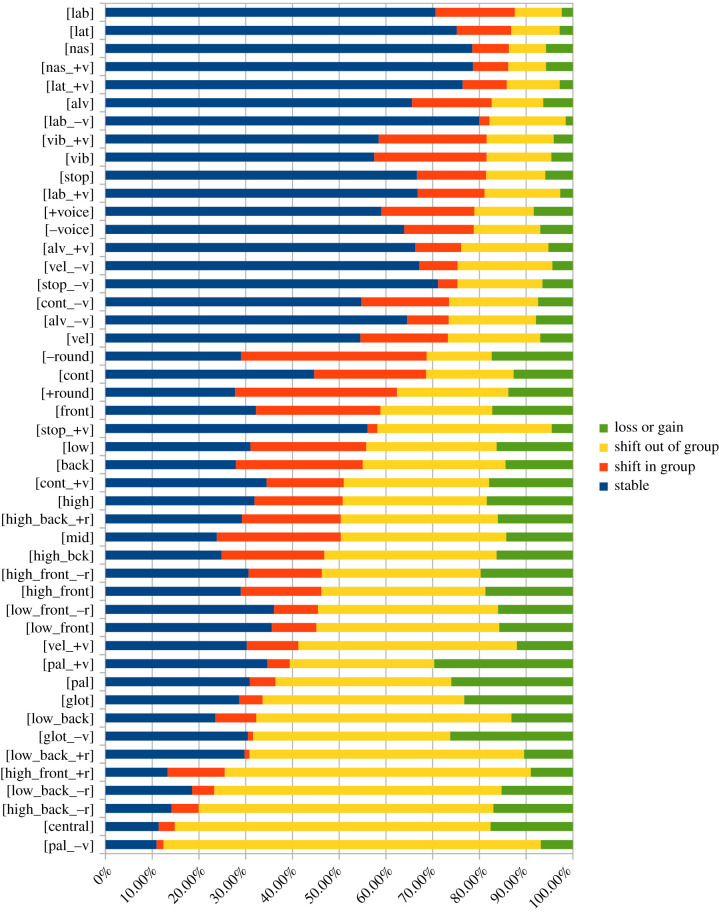
Barplot demonstrating the stability rates of sound groups, divided by ‘stable’ and ‘shift in group’ (=stability rate) versus ‘shift out of group’ and ‘loss or gain’ (=instability rate), organized from most stable sound group (top) to most unstable (down) (see electronic supplementary material, S1).

If we begin with the sound stability values alone (SSt; electronic supplementary material, S1), the result distinguishes four types: (i) stable (i.e. the percentage with an exact matching in homologues), (ii) shift in group (i.e. the percentage with a matching within the sound group), (iii) shift out of group (i.e. the percentage with a matching outside of the sound group) and (iv) loss or gain (i.e. the percentage of cases where a phoneme is lost or gained). We summarize these into two types, *stable* (stable + shift in group) and *unstable* (shift out of group + loss or gain) (figure 2). We conclude that the stability values of sound groups are highly diverging, from almost complete stability (labials, laterals, nasals) to almost complete instability (central vowels, palatals, glottals). The most striking result is the difference in stability between consonants and vowels, where consonants have higher stability values.

**Figure 3.**
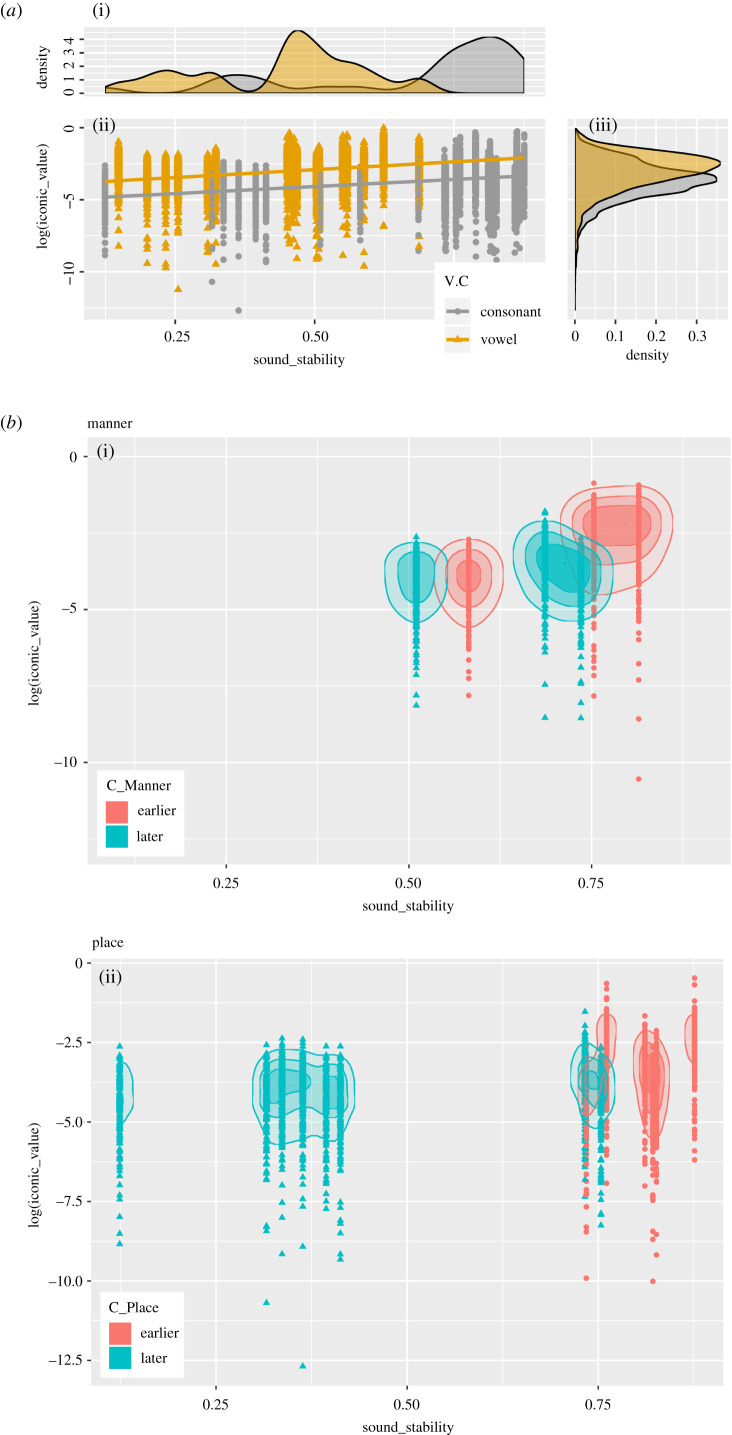
(*a*) Density plot (i) and scatter plot with regression line (ii) with a corresponding density plot (iii), adapted to a logarithmic scale, comparing sound stability (x) and iconic value (y), separating the distribution of vowels (yellow) and consonants (grey) (see electronic supplementary material, S4, S5). (*b*) Density scatterplots, adapted to a logarithmic scale, comparing sound stability (x) and iconic value (y), separating the distribution of ‘earlier’ (red) and ‘later’ (blue) in first language acquisition (relative distinctions of acquisition order of features, following the babbling period), given by the feature classes ‘manner of articulation’ (i) and ‘place of articulation’ (ii) (see electronic supplementary material S4-S6). (Online version in colour.)

Second, we correlate the rates of SSt (stable + shift in group) to the Ico, separating consonants and vowels. We use a density plot and a scatter plot adapted to a logarithmic scale for a better visualization of the results (figure 3*a*). The density plot shows two clear density peaks for SSt and Ico, one for consonants (with high stability) and one for vowels (with medium stability). Three linear regression tests (electronic supplementary material, S5, S6) show that separating vowels and consonants leads to a better model, as iconic value appears to be systematically higher for vowels than for consonants. The linear regression analysis involving both SSt and Ico is significant when looking at vowels only (*F*_1,2770_ = 108.7, *p* < 0.001, *R*^2^ = 0.03777), consonants only (*F*_1,4119_ = 398.6, *p* < 0.001, *R*^2^ = 0.08823) and all data (*F*_1,6891_ = 246.08, *p* < 0.001, *R*^2^ = 0.006642). The larger *R*^2^ values for the vowels-only and consonant-only models indicate that the separation explains a larger portion of the variance in the data, compared to an all-data model. On the other hand, the relatively low *R*-values indicate that there is a large degree of variance in the data. As expected, the effect of iconicity, in terms of variance explained, is relatively small, but statistically significant. Iconicity is not a main driving force of sound evolution, but factors in a small, but steady way.

Finally, we contrast SSt and Ico, separating earlier and later in L1, extracting rows with sound groups that have been coded for this feature (electronic supplementary material, S4, S5). We find that there is a clear separation by place of articulation, where earlier is higher in stability and iconic value (figure 3*b*ii). Manner of articulation shows the same pattern, but with a bimodal separation (figure 3*b*i). Voicing, as well as vowels (openness/backness), cannot be as clearly separated into earlier and later with respect to SSt and Ico (electronic supplementary material, S6).

## 5. Discussion

Our results should be considered in relation to our theory as well as earlier literature [4,5,13]. Apparently, vocal iconicity is overrepresented with sound groups of the higher stability spectrum, both for consonants and vowels, but with an internal difference: high for consonants and moderately high for vowels (figure 3*a*). Even though vowels are generally more unstable (figure 2), the iconic value is overall higher for vowels (figure 3*a*). This means that our assumption about the correlation between sound stability and iconicity generally holds for both consonants and vowels. The pattern recurs when we match our data with observations of earlier and later in L1. Place and manner of articulation confirm the predictions that stability and iconicity co-occur with the sound group types that are acquired earlier when learning a language (figure 3*b*). However, the pattern by vowels gives no indication, which is interesting and noteworthy considering the fact that vowels as such are more profiled in sound–meaning mappings (electronic supplementary material, S6).

The results give rise to several questions which cannot be fully answered by our study. The causality of the observed patterns is not entirely clear, and there are several possible explanations for pre-historic language. Vocal iconicity may be an effect of certain high-stability sound groups randomly occurring more in words for some concepts, which would then appear iconic because they are more resistant to change. Alternatively, only high-stability sound groups survive in iconic mappings because they reflect the way in which words were coined when language evolved, but the iconic low-stability sounds did not survive until the present day. It is also possible that there is a bias for sound laws, or sporadic changes, to favour stabilizing iconic form–meaning mappings. Our comparison with L1 order indicates that children acquiring language trigger iconic mappings, at least for the consonantal onset (and possibly the coda) of the syllable, but not for the vowel core, which on the other hand is more frequent in iconic mappings. This indicates that the L1 theory, even though it is supported by our results, cannot completely explain the evolution and emergence of vocal iconicity.

## 6. Conclusion

Based on a dataset of 107 Eurasian languages, we find that preference in speech production matches sound stability in a relatively straight-forward way, which follows mechanisms such as the sonority hierarchy and place and manner of articulation. Sonorous vowels are the most unstable sounds; continuants, vibrants and laterals show a steadily increasing stability; alveolar and dental stops are even more stable; and labials, nasals and laterals are the most stable sounds. Vocal iconicity, i.e. resemblance-based form–meaning mapping of specific concepts, measured on a dataset of global coverage, is generally restricted to sound groups, which are higher in the sound stability spectrum. This goes for both consonants and vowels, where a separation of consonants and vowels considerably improves the result. This indicates that iconic sound–meaning mappings are more likely to survive sound evolution and change compared to an average arbitrary connection between form and meaning. For consonants, specifically in the feature classes place and manner of articulation, sound groups of high-stability and high iconic value co-occur with sound groups that are acquired earlier in L1. There is no such pattern for vowels, even though vowels are generally more frequent in iconic mappings. This indicates that for consonants, children acquiring language trigger form–meaning mappings and reinforce cross-linguistic patterns over time.

## Footnotes

1 See https://github.com/jdellert/icon-evol.

## Data accessibility

Two of the three relevant input datasets (NorthEuraLex and the Iconicity values) were previously published and are openly accessible (http://www.northeuralex.org/, https://osf.io/dh7f3/). The third input dataset (L1 values) as well as our combined dataset which was used for all analyses are available both as electronic supplementary materials (S2–S4) and via our GitHub repository (https://github.com/jdellert/icon-evol), which also hosts all of the relevant code.
